# *Wolbachia* genetically interacts with the *bag of marbles* germline stem cell gene in male *D. melanogaster*

**DOI:** 10.17912/micropub.biology.000845

**Published:** 2023-05-25

**Authors:** Miwa Wenzel, Charles F. Aquadro

**Affiliations:** 1 Molecular Biology and Genetics, Cornell University, Ithaca, New York, United States

## Abstract

The bacterial endosymbiont *Wolbachia* manipulates reproduction of its arthropod hosts to promote its own maternal vertical transmission. In female *D. melanogaster* , *Wolbachia* has been shown to genetically interact with three key reproductive genes ( *bag of marbles* ( *bam* ) *, Sex-lethal,* and *mei-P26)* , as it rescues the reduced female fertility or fecundity phenotype seen in partial loss-of-function mutants of these genes *.* Here, we show that *Wolbachia* also partially rescues male fertility in *D. melanogaster* carrying a new, largely sterile *bam* allele when in a *bam* null genetic background. This finding shows that the molecular mechanism of *Wolbachia* ’s influence on its hosts' reproduction involves interaction with genes in males as well as females, at least in *D. melanogaster* .

**Figure 1. Fertility and cytology of male Drosophila melanogaster:**
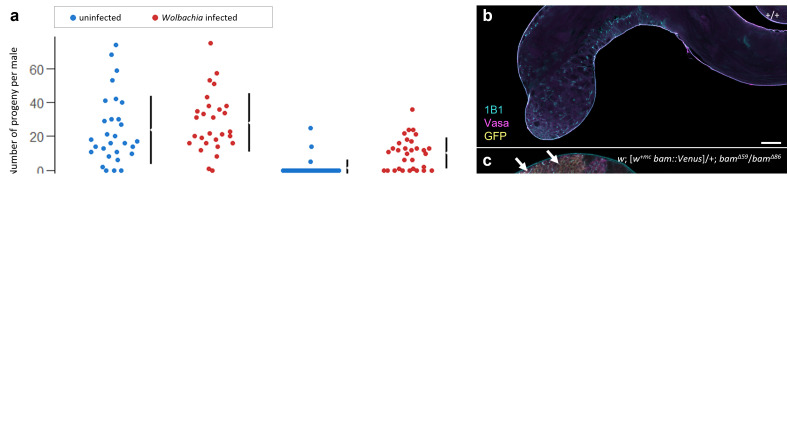
**(a)** Jitter plots (top) display the number of progeny per male fly over seven days after the first day of progeny eclosion, with data from uninfected flies in blue and *Wolbachia-* infected flies in red. The mean differences between the uninfected and infected flies are plotted below as a black dot with 95% confidence intervals represented by the black vertical line. A two-sided permutation t-test comparing the uninfected and infected samples resulted in p=0.409 and p=0.0 for transgenic *bam* ( *w; [w ^+mc^ bam]; bam ^Δ59^ /bam ^Δ^* ^86^ ) and transgenic *bam::Venus* ( *w; [w ^+mc^ bam::Venus]; bam ^Δ59^ /bam ^Δ86^* ) *,* respectively. **(b-d)** Wildtype and mutant *D. melanogaster* testes stained with Hts-1B1, Vasa, and GFP. **(b)** Wildtype CantonS (+/+), **(c)** transgenic *bam::Venus* ( *w; [w ^+mc^ bam::Venus]; bam ^Δ59^ /bam ^Δ86^* ), **(d)** endogenous *bam::Venus* ( *w; bam::Venus/bam ^null-In2-3xP3-DsRed^ )* . Note the small, tumorous cyst cells in (c) as indicated by the arrows that are present in the transgenic, but not in (d) the endogenous *bam::Venus* testis *,* despite both having only one copy of functional *bam* . Scale bar for all images is 50μM. Hts-1B1 identifies fusomes, Vasa identifies germ cells, and GFP identifies Bam::Venus protein.

## Description

The widespread bacterial endosymbiont *Wolbachia* is well known for its manipulation of host reproduction across arthropods. This includes rescue of fertility defects observed in partial loss-of-function mutants in *D. melanogaster* females, with genes including *bag of marbles* ( *bam* ) (Flores et al. 2015, Bubnell et al. 2021) , *Sex-lethal* ( *Sxl* ) (Starr and Cline 2002, Ote et al. 2016), and *meiotic P26* ( *mei-P26* ) (Russell et al. 2022) .

*Bam* is a germline stem cell (GSC) gene critical in female and male *D. melanogaster* where it regulates cell maintenance and differentiation. In females, *bam* functions primarily as a key switch gene that initiates the differentiation programming of GSC daughter cells into cystoblasts (McKearin and Spradling 1990) . In males, *bam* functions to limit the mitotic divisions of spermatogonia and initiate programming of spermatocytes (McKearin and Spradling 1990, Gonczy 1997, Insco et al. 2009, Insco et al. 2012) . In spermatogonia, Bam protein accumulates to a threshold that transitions the cells into spermatocytes. There, Bam is quickly downregulated and the translation of *bam* mRNAs is repressed by microRNAs (Eun et al. 2013) .

It was previously shown that *Wolbachia* genetically interacts with *bam* in female *D. melanogaster* but not male *D. melanogaster* (Flores et al. 2015) *.* However, because the *bam* mutant tested was sterile in males, it is unknown if the mutation is too severe for *Wolbachia* to rescue the fertility defect or if *Wolbachia* does not genetically interact with *bam* in males. For the former case, this possibility is evident in the lack of rescue of a *bam* null mutant (Flores et al. 2015) . For the latter case, *Wolbachia* may not genetically interact with *bam* in males because of *bam* ’s differing function between the sexes.

We have found a *bam* allele that has reduced fertility in males when expressed as a transgene in a *bam* null background, thus allowing us to distinguish between the two possibilities *.* The *bam* allele is a PhiC31 generated transgene inserted at the attP40 site on the second chromosome and expresses a fluorescent Venus-tagged Bam protein. These flies have a severe fertility defect in a sensitized *bam* null background when uninfected with *Wolbachia* that is not seen in the transgenic *bam* line that lacks the Venus fluorescent tag in a similar *bam* null background (p<0.05) (Fig 1a).

We find that the reduced fertility of the transgenic *bam::Venus* males is rescued by *Wolbachia* (Fig 1a) (p<0.05). In contrast, the transgenic *bam* line without the Venus tag is not affected by *Wolbachia* infection. These results show that *Wolbachia* genetically interacts with *bam* in male *D. melanogaster* , as it does in female *D. melanogaster.*

We compared the cytology of this transgenic *bam::Venus* line to that of a single copy of an endogenous *bam::Venus* line that was generated with CRISPR (Bubnell 2020) . The transgenic *bam::Venus* males displayed tumorous cells that continued to express Bam protein (Fig 1c). The GSC population in the testis hub appeared to remain intact with no obvious ectopic Bam expression in the stem cell niche. In contrast, the endogenous *bam::Venus* testes did not show a severe tumorous phenotype, though other cytological defects are observed, as *bam* is haploinsufficient in males and *D. melanogaster* testes require two copies of *bam* to be cytologically wildtype (Fig 1d) (Insco et al. 2009) . These results suggest that the tumorous phenotype in the transgenic *bam::Venus* testes is due to the transgenic nature of the allele, wherein the genomic location of the transgene insert is likely affecting *bam* expression (Spradling and Rubin 1983, Levis et al. 1985) . This is consistent with findings that reproduction is sensitive to *bam* expression levels in male *D. melanogaster* , as shown through cytological assays in Insco et al. (2009) and in a *bam* intron disruption line (Bubnell 2020) .

Two lines of reasoning could explain *Wolbachia* ’s fertility rescue of our male hypomorphic mutant. The first is that it is evolutionarily advantageous for *Wolbachia* to inhabit a reproductively fit male host. *Wolbachia* spreads rapidly through populations by inducing cytoplasmic incompatibility, where *Wolbachia* manipulates host sperm (reviewed in Wang et al. 2022). Thus, it is not unreasonable to consider that, despite being maternally inherited, *Wolbachia* that help maintain proper spermatogenesis have been evolutionarily favored.

The second line of reasoning considers similarities in the germline of female and male *D. melanogaster* hosts. *Wolbachia* may interact with host proteins or RNA that function similarly in oogenesis and spermatogenesis. Thus, *Wolbachia* ’s genetic interaction with *bam* in males may be a byproduct of *Wolbachia* ’s actions in females that help maintain proper oogenesis to promote *Wolbachia* ’s survival through maternal transmission. In this vein, it is worth noting the recent report that *Wolbachia* also rescues the fertility defect of a largely sterile male *mei-P26* null mutant (Russell et al. 2022) , where *mei-P26* is a gene critical for both male and female gametogenesis (Page et al. 2000; Neumuller et al. 2008) .

Although *bam* ’s primary function is different in male and female *D. melanogaster* , there are some commonalities between males and females that we highlight here. 1) *bam* null mutants produce tumorous cells that display GSC-like qualities (in males, the tumorous cells also display some qualities of germ cells, such as incomplete cytokinesis and synchronic mitosis) (McKearin and Ohlstein 1995, Gonczy et al. 1997) . 2) Ectopic Bam expression in GSCs can cause GSC loss (Ohlstein and McKearin 1997, Shulz et al. 2004, Kawase et al 2004, Flatt et al. 2008, Sheng et al. 2009) . 3) Partially differentiated germ cells are able to dedifferentiate back into GSC-like cells with manipulation of *bam* (Kai and Spradling 2004, Sheng et al. 2009) . And 4) *bam* may share a role of limiting mitotic divisions: it has been proposed that degradation of Bam after the fourth mitotic division prevents further mitosis in females (McKearin and Ohlstein 1995) and it has been shown in males that accumulation of Bam to a threshold stops the mitotic programming of spermatocytes (Insco et al. 2009) .

Our current results do not allow us to distinguish between these two lines of reasoning to explain *Wolbachia* ’s fertility rescue of a male hypomorphic mutant and more research on *bam* function and its molecular partners in male *D. melanogaster* is needed before we are able to get a clear understanding of the similarities and differences in how *Wolbachia* interacts with *bam* in females and males *.* Nevertheless, establishing a genetic interaction between *bam* and *Wolbachia* in male *D. melanogaster* is an important insight into the functional interaction between *Wolbachia* , *bam* , and gametogenesis.

## Methods

**Fly lines.** Two lines of transgenic flies were generated and examined for fertility defects in male *D. melanogaster* . These include the *w* ; [ *w ^+mc^ bam::Venus* ] and a *w* ; [ *w ^+mc^ bam* ] line that lacks the Venus fluorescent tag. The transgenic alleles were cloned and mutant flies were generated by PhiC31 transgenesis as described in detail in Wenzel and Aquadro (2023). Briefly, the entire *bam* coding region, its introns, and approximately 1.5kb upstream and 700bp downstream were cloned from the genomic DNA of a *bam::Venus* line (Bubnell 2020) . The design of this construct is based on the *D. melanogaster bam* YFP line from Flores et al. (2015), but contains a 36bp glycine-glycine-serine linker sequence between the 3’ end of the *bam* coding sequence and the Venus tag to promote proper protein folding. The *bam::Venus* construct was cloned into the pCasper\attB vector, a gift from Dan Barbash, and inserted at the attP40 site on the second chromosome, with plasmid injections performed by GenetiVision into the embryos of a *w, nos-* int *;* P{CaryP}attP40 *D. melanogaster* line.

Transgenic flies that were assessed for this study were in a null *bam* background such that the genotypes are *w* ; [ *w ^+mc^ bam::Venus* ]/+; *bam ^Δ59^ /bam ^Δ86^* and *w* ; [ *w ^+mc^ bam* ]/+; *bam ^Δ59^ /bam ^Δ86^ . bam ^Δ59^* and *bam ^Δ86^* were originally obtained from Dennis McKearin. The *w* Mel strain of *Wolbachia* was maternally crossed in from PCR-verified infected parental strains when appropriate during generation of the desired genotypes.

Gene edited flies that were used in this study include *w; bam::Venus/bam ^null-In2-3xP3-DsRed^* , where, here, *bam::Venus* is an CRISPR induced Venus-tagged endogenous *bam* allele (Bubnell 2020) and *bam ^null-In2-3xP3-DsRed^* is an intron disruption null allele (Bubnell 2020; Bubnell 2021) .

**Fertility assays.** Virgin transgenic males and virgin CantonS females were collected and aged four days at which time they were mated to each other in individual crosses. At least 29 crosses of one male and two females were set up on yeast-glucose food (minimum of 29, maximum of 40 crosses were set per genotype tested). All crosses were kept in an incubator at 25°C with a 12-h light/dark cycle. Parents were removed on the seventh day of mating. Progeny were counted every other day over seven days, starting on the day of first eclosion.

To offer greater transparency of data, estimation statistics and plots were generated using the dabestr package in R (v0.3.0) (Ho et al. 2019). The associated estimation statistics website was used to report p-values from two-sided permutation t-tests, as they were not provided in the R package (Claridge-Chang and Ho, accessed 2022 Sept 20).

**Immunostaining.** Immunostaining was performed on testes of 2-3 day old transgenic flies as described in Flores et al. (2015) and Bubnell et al. (2022). Briefly, testes were dissected in ice-cold 1x PBS and fixed in 4% paraformaldehyde (Electron Microscopy Sciences) for 15 min. Tissue was subsequently rinsed and washed with PBST (1X PBS, 0.1% Triton-X100), blocked in PBTA (1X PBS, 0.1% TritonX100, 3% BSA) (Alfa Aesar), and incubated overnight with primary antibodies. Rinse and wash steps were repeated with PBTA, followed by an overnight incubation with goat anti-serum, and a 2 hour incubation with secondary antibodies. Tissue was rinsed and washed again with PBST and mounted in ProLong Diamond AntiFade (Invitrogen).

Primary antibodies used include anti-Vasa (anti-rat, 1:20, Developmental Studies Hybridoma Bank (DSHB)), anti-Hts-1B1 (anti-mouse, 1:40, DSHB), and anti-GFP (anti-rabbit, 1:200, Invitrogen Cat No: G10362). All secondary antibodies were used at 1:500, including goat anti-rat Alexa488 (Invitrogen Cat No: A-11006), goat anti-mouse Alexa568 (Invitrogen Cat No: A-11031), and goat anti-rabbit Alexa633 (Invitrogen Cat No: A-21070). Tissue was imaged on a Zeiss i880 confocal microscope with 405, 488, and 568nm laser lines at 63X (Plan-Apochromat 1.4 NA, oil) (Cornell BRC Imaging Core Facility). Images were edited using Fiji (Schindelin et al. 2012) .
